# MMP1 acts as a potential regulator of tumor progression and dedifferentiation in papillary thyroid cancer

**DOI:** 10.3389/fonc.2022.1030590

**Published:** 2022-11-21

**Authors:** Jun Zhou, Ming Xu, Jie Tan, Lin Zhou, Fang Dong, Tao Huang

**Affiliations:** Department of Breast and Thyroid Surgery, Union Hospital, Tongji Medical College, Huazhong University of Science and Technology, Wuhan, China

**Keywords:** papillary thyroid cancer, poorly differentiated thyroid cancer, anaplastic thyroid carcinoma, dedifferentiation, MMP1

## Abstract

Papillary thyroid cancer (PTC) is one of the malignancies with an excellent prognosis. However, in PTC, progression or dedifferentiation into poorly differentiated thyroid cancer (PDTC) or anaplastic thyroid cancer (ATC) extremely jeopardizes patients’ prognosis. MMP1 is a zinc-dependent endopeptidase, and its role in PTC progression and dedifferentiation is unclear. In this study, transcriptome data of PDTC/ATC and PTC from the Gene Expression Omnibus and The Cancer Genome Atlas databases were utilized to perform an integrated analysis of MMP1 as a potential regulator of tumor progression and dedifferentiation in PTC. Both bulk and single-cell RNA-sequencing data confirmed the high expression of MMP1 in ATC tissues and cells, and further study verified that MMP1 possessed good diagnostic and prognostic value in PTC and PDTC/ATC. Up-regulated MMP1 was found to be positively related to more aggressive clinical characteristics, worse survival, extracellular matrix-related pathways, oncogenic immune microenvironment, more mutations, higher stemness, and more dedifferentiation of PTC. Meanwhile, *in vitro* experiments verified the high level of MMP1 in PDTC/ATC cell lines, and MMP1 knockdown and its inhibitor triolein could both inhibit the cell viability of PTC and PDTC/ATC. In conclusion, our findings suggest that MMP1 is a potential regulator of tumor progression and dedifferentiation in PTC, and might become a novel therapeutic target for PTC, especially for more aggressive PDTC and ATC.

## Introduction

Papillary thyroid cancer (PTC) accounts for more than 80-85% of all thyroid cancers (TCs) (1), and PTC patients usually have a 10-year survival rate greater than 90% (2). Most PTC responds well to the current treatments, including surgery, thyroid-stimulating hormone suppression, and radioactive iodine therapies (2). However, 10-15% PTCs eventually experience recurrence or metastasis, dedifferentiate into more aggressive poorly differentiated TCs (PDTCs), and develop treatment resistance, consequently leading to cancer-related mortality (3–5). It was reported that the 5-year survival rate of PDTC patients was only 50-64% (6, 7), leaving large gaps with other PTC patients. Anaplastic thyroid cancer (ATC) is an undifferentiated TC that accounts for only 1-2% of all TCs. As one of the most lethal malignancies in humans, ATC has cancer-specific mortality at one year of nearly 100%, and it often originates from a pre-existing presence of differentiated thyroid cancer (DTC) including PTC or occurs *de novo* (2, 8, 9). Therefore, it is clinically important to precisely stratify the aggressive PTC for active intervention to avoid its progression and dedifferentiation into PDTC/ATC.

Clinical variables of PTC, including the tumor node metastasis (TNM) stage, are routinely applied to clarify the mortality or recurrence risks of PTC (2). Nevertheless, despite their role in treatment selections, these factors remain insufficient to predict tumor progression after surgical treatment, especially the potential for dedifferentiation. Recent genomic studies on PDTC and ATC provided deep insights into the molecular pathogenesis and facilitating tumoral progression from PTC to PDTC/ATC through the accumulation of crucial genetic alterations, such as *BRAF* and *TERT* mutations (10–13). Although these findings help assess subtyping, prognostication, and therapy, while some targeted therapies are effective in a small fraction of PDTC/ATC patients (14), the deeper molecular mechanisms of PTC progression and dedifferentiation are only partially explained. It remains challenging to identify novel biomarkers, as well as potential therapeutic targets for further research.

MMP1, a zinc-dependent endopeptidase, is a member of matrix metalloproteinases (MMPs) and has been reported to be involved in multiple biochemical mechanisms in cardiovascular renal disorders, inflammation, and malignancy (15). Currently, very few studies have comprehensively explored the role of MMP1 in PTC and PDTC/ATC. Recent three studies indicated that MMP1 expression levels positively correlated with higher clinical stages of PTC (16–18), but its association with differentiation level and prognosis remains unclear. Paul Weinberger et al. also reported up-regulated MMP1 in ATC (19), but no further studies have been carried out.

In the present study, we screened the Gene Expression Omnibus (GEO) datasets to discover differentially expressed genes (DEGs) between PDTC/ATC and PTC, and then the role of MMP1 in PTC progression, immune infiltration, mutation, stemness, and differentiation was further discovered and validated using The Cancer Genome Atlas (TCGA) cohort *via* integrated bioinformatics analysis. Our results demonstrate the clinical utility of MMP1 and the implicational potential as a biomarker for PTC and PDTC/ATC, and the *in vitro* functional experiments confirmed its role as a potential therapeutic target.

## Methods and materials

### Study design and data source

**Figure 1:**
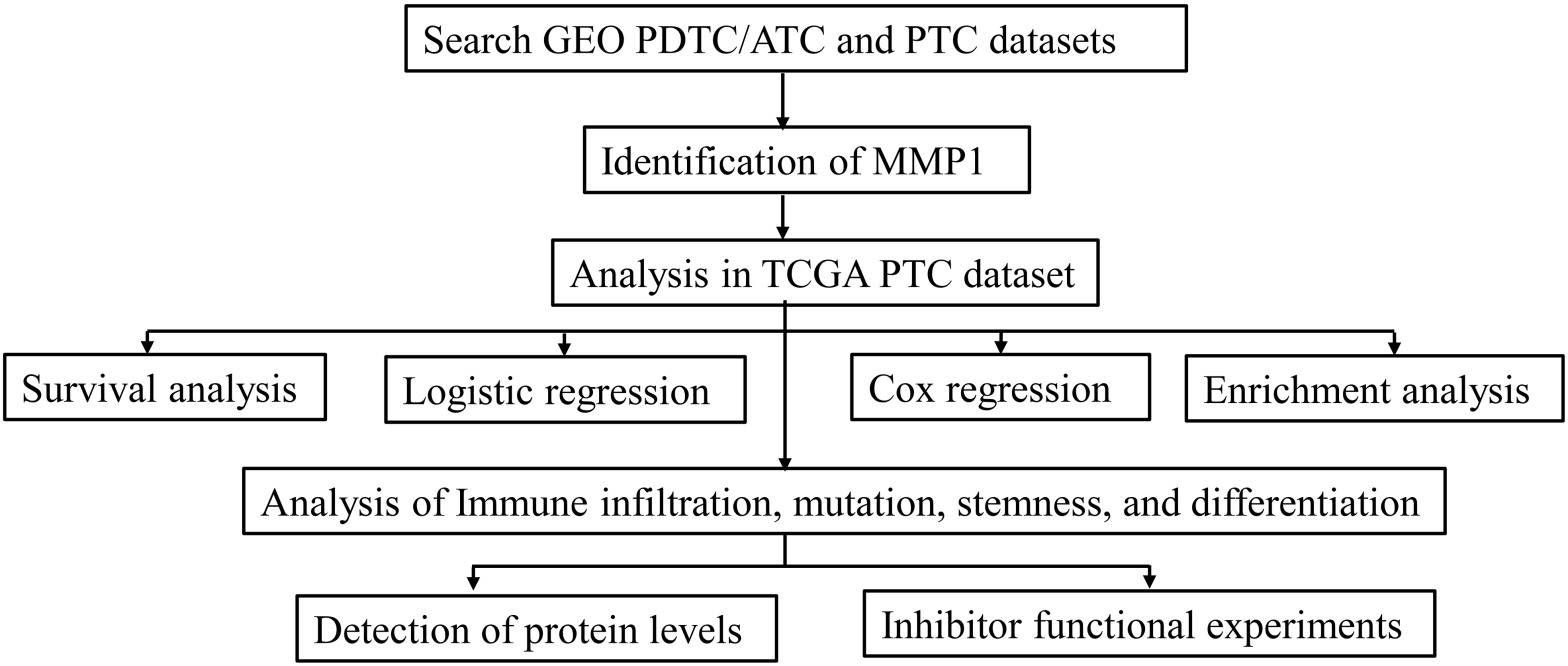
Flow diagram of this study. The details of data collection and analysis were exhibited in a flow diagram. GEO, the Gene Expression Omnibus; PDTC, poorly differentiated thyroid cancer; ATC, anaplastic thyroid cancer; PTC, papillary thyroid cancer; TCGA, The Cancer Genome Atlas.

The process of this study is shown in the flow chart (**Figure 1**). The gene expression data and clinicopathological data were obtained from TCGA (https://www.cancer.gov/tcga) and GEO (https://www.ncbi.nlm.nih.gov/geo/) databases. Multiple analyses were performed using R (http://www.r-project.org, version 4.1) in this study. Sequencing data from different GEO datasets were batch-normalized using the “sva” package (20) and subsequently analyzed. The GEO accession codes of microarray data were all base on an identical platform (Affymetrix Human Genome U133 Plus 2.0 Array) and utilized in this study (GSE76039, GSE66030, GSE29265, GSE53157, and GSE65144). PDTC and ATC samples from GEO were pooled together in cross-comparison with PTC samples. Clinical information of 37 PDTC/ATC patients from GSE76039 was downloaded from cBioPortal (http://www.cbioportal.org/) (21). The median RNA sequencing value was chosen as the cut-off value of the cohorts included in this study.

### Identification of DEGs and MMP1

The R package “limma” (22) was used to screen DEGs with the adjusted P-value <0.05. The “umap” package (23) was used to perform the sample heterogeneity analysis between PTC and PDTC/ATC. The “ComplexHeatmap” package (24) was used for customizing the heatmap and the “ggplot2” package (25) was used for visualization.

### Single-cell RNA sequencing analysis

We downloaded the original single-cell RNA sequencing data from five ATC patients and six PTC patients on the GEO database (GSE148673 and GSE191288). After standard data quality control, batch effect adjustment, and normalization using the “Seurat” package (26), we clustered all cells using the Uniform Manifold Approximation and Projection (UMAP) method (27) as four basic types: tumor/epithelial cells, immune cells, endothelial cells, and fibroblasts *via* cell markers (**Supplementary Figure 1A**). MMP1 expression was analyzed in PTC/ATC and all kinds of cells.

### Correlation analysis

The “DESeq2” and “corrplot” packages (28, 29) were used to perform a spearman correlation analysis between the expression levels of MMP1 and other different indicators, and a P-value <0.05 was selected as a cutoff criterion. The receiver operator characteristic (ROC) curve analysis was performed using the “pROC” package (30) to evaluate the diagnostic value of MMP1. Logistics regression and Cox regression with 95% confidence intervals (CIs) were performed in R to calculate the odds ratios (ORs) and hazard ratios (HRs) to assess the value of MMP1 in predicting some pathological characteristics and outcomes of PTC patients from TCGA, respectively,

### Survival analysis

The survival data were obtained from the TCGA-THCA dataset. Considering the favorable prognosis of most PTC patients, the correlation between the number of death events and overall survival (OS) was pretty low, and we focused on the progression-free survival (PFS) and disease-free interval (DFI) of the patients. The median of MMP1 expression was defined as the cutoff point for dividing the samples into high and low-expression groups. The survival probability was estimated *via* Statistical Product and Service Solutions (SPSS, version 26.0) using the Kaplan-Meier method, and a log-rank P-value <0.05 was considered statistically significant. Data from Gene Set Cancer Analysis (GSCA) database (31) (http://bioinfo.life.hust.edu.cn/GSCA/#/) were also adopted into survival analysis. Although there is a difference in the prognosis between ATC and PDTC that we cannot ignore, their outcomes are still very poor compared with PTC. Referring to the study (32) of Wen et al., we pooled ATC and PDTC from GSE76039 together to analyze the relationship between their prognosis and MMP1 level. Cox proportional hazard regression model was used to assess the survival difference and hazard ratio (HR) of MMP1 in PTC patients.

### Enrichment analysis

Enrichment analysis has been widely used in recent years to identify gene properties, based on the hypothesis that genes with similar expression profiles may be regulated by common pathways and involved in related functions (33). In this study, Gene Ontology (GO), Kyoto Encyclopedia of Genes and Genomes (KEGG) enrichment analysis, and Gene set enrichment analysis (GSEA) were performed in different sample groupings to find potential molecular mechanisms. The “clusterProfiler” package (34) was used, and nominal P <0.05 and false discovery rate (FDR) <0.25 were selected as cutoff criteria.

### Immune infiltration analysis

Multiple gene set signature-based methods for comprehensively estimating the abundance of different immune cell types were utilized in this study. To estimate the variation of involved immune cells over PTC samples, a gene set enrichment analysis called Gene Set Variation Analysis (GSVA) was performed (35). The “ESTIMATE” package (36) was applied to calculate the immune scores of each PTC sample, and the correlation between immune cell infiltrate and the GSVA enrichment score of PTC was further verified. The immune infiltration landscape of PTC was also conducted *via* the “ssGSEA” algorithm in the “GSVA” package (37), “TIMER” and “xCELL” algorithms in the “IOBR” package (38, 39) to identify immune cells that might be significantly associated with MMP1.

### Analysis of the mutation, stemness, and differentiation

The stemness level of a tumor could be quantified based on the RNA expression and DNA methylation signature (40), and the Epigenetically regulated RNA expression-based Stemness Score (EREG.EXPss, 103 probes) and DNA methylation-based Stemness Score (DNAss, 219 probes) were utilized in this study to assess the stemness of PTC from TCGA. *BRAF* ^V600E^-*RAS* score (BRS) was developed by the TCGA group to quantify the extent to which the gene expression profile of PTC resembles either the *BRAF* ^V600E^- or *RAS*-mutant profiles (41). Thyroid differentiation Score (TDS) was also developed by the TCGA group to quantify the PTC differentiation level (41) and it was calculated based on the expression levels of 16 thyroid metabolism and function genes (DIO1, DIO2, DUOX1, DUOX2, FOXE1, GLIS3, NKX2-1, PAX8, SLC26A4, SLC5A5, SLC5A8, TG, THRA, THRB, TPO, TSHR). Data of stemness scores, BRS, and TDS were obtained from TCGA and their relationships with MMP1 in PTC were also further explored.

### Cell culture

The PTC cell lines TPC-1 and K1, PDTC cell line KTC-1, and ATC cell line CAL-62 used in this study were purchased from the American Type Culture Collection (ATCC). All cells were cultured in the 37°C and 5% CO_2_ culture environment, and in specific mediums (Gibco, USA) suggested by ATCC with 10% fetal bovine serum (FBS).

### Western blotting analysis

The total protein of cells was extracted with protein extraction reagent Radio-immunoprecipitation Assay buffer (Beyotime, China) containing 1mM Phenylmethylsulfonyl fluoride (Beyotime, China), and quantified by the BCA Protein Assay Kit (Beyotime, China). Equal amounts of protein were subjected to 10% SDS-PAGE and then transferred to a PVDF membrane (Millipore, USA). The immunoblots were incubated with primary antibodies against MMP1 (1: 800 dilution, Proteintech, China), and GAPDH (1: 3000 dilution, Cell Signaling Technology, USA) as the internal control. The protein signals were visualized with the ChemiDoc XRS+ System (Bio-Rad, USA) using the ECL detection kit (Beyotime, China).

### Cell transfection

Short interference RNA (siRNA) for MMP1 and corresponding siRNA negative control were purchased from RiboBo (China). Transient transfection was performed using Lipofectamine 3000 (Thermo Fisher, USA) according to the manufacturer′s protocol. The transfection efficiency was evaluated using western blot and the siRNA target sequence for MMP1 was as follows: 5′- ACACAAGAGCAAGATGTGG-3′; The cells were harvested at 48 hours after transfection and then used for further experiments.

### Cell viability assays

The cell viability assays were analyzed by the Cell Counting Kit-8 (CCK8) and colony formation assay to evaluate the cell proliferation of different cells according to the manufacturer’s protocol.

CCK8 assay: A total of 1000 cells were seeded into 96-well plates with different concentrations of MMP1 inhibitor Triolein (42). After 48 hours, the medium was removed and a 100 μL serum-free medium with 10% CCK8 solution (Dojindo, Japan) inside was added to each well of the plate. After incubation for 2 hours at 37°C, the spectrometric absorbance of each well at 450 nm was measured on a microplate reader (Thermo Fisher, USA).

Colony formation assay: A total of 1000 cells were seeded into 6-well plates with 50 μM Triolein. All cells were cultured in their corresponding medium, and the medium was renewed every three days over the next 15 days. Cell clones were stained with 0.2% crystal violet (Beyotime, China) and then photographed.

### Statistical analysis

SPSS 26.0 (IBM, USA), R 4.1 (Lucent Technologies, USA), and Graphpad Prism 8.0 (GraphPad Software, USA) were used for statistical analysis. ImageJ 1.53k (NIH, USA) was used for colony formation counting. Unless stated otherwise, the Student’s t-test, two-way analysis of variance, or Chi-square test was performed to compare the differences between different groups, respectively. Results of P <0.05 were considered significant: NS means not significant, *P <0.05, **P <0.01, ***P <0.001, and ****P <0.0001.

## Results

### Identification of MMP1 between PTC and PDTC/ATC

**Figure 2:**
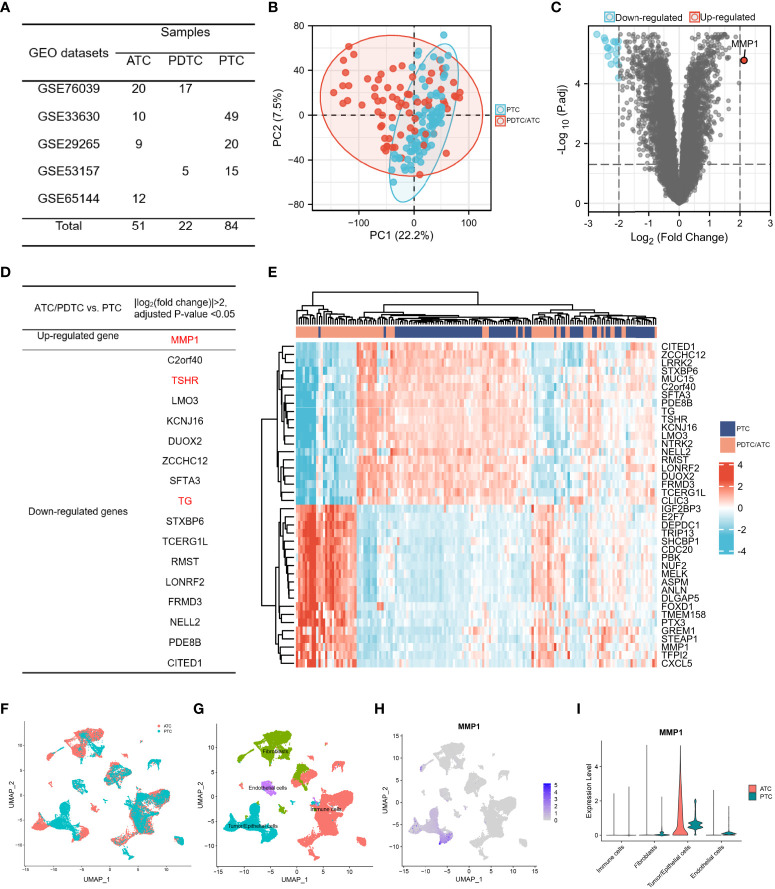
The identification of MMP1. **(A)** TC samples from the GEO database included in this study; **(B)** PCA map between PDTC/ATC and PTC. The difference between PTC and PDTC/ATC samples was not very large. **(C)** Volcano plots exhibited the DEGs between PDTC/ATC and PTC. **(D)** List of DEGs between PDTC/ATC and PTC. **(E)**. Heat map of MMP1 and another top 40 DEGs between PDTC/ATC and PTC. **(F)** Single-cell sequencing data in five ATC and six PTC patients. **(G)** Four basic types of cells in single cell sequencing data: tumor/epithelial cells, immune cells, endothelial cells and fibroblasts. **(H)** MMP1 expression in different cells. **(I)** Relative quantification of MMP1 expression level. GEO, the Gene Expression Omnibus; PDTC, poorly differentiated thyroid cancer; ATC, anaplastic thyroid cancer; PTC, papillary thyroid cancer; DEG, differentially expressed gene.

Bulk gene expression data were extracted from 5 GEO datasets and samples were listed in **Figure 2A**. 73 advanced thyroid tumors (22 PDTCs and 51 ATCs) and 84 PTCs met the sequencing quality standards and are included in this study. The sample-to-sample heterogeneity between PDTC/ATC and PTC was detected by principal component analysis (PCA) (43)(**Figure 2B**). 351 DEGs between these two groups were screened (Details in **Supplementary Table 1**) while the cutoff criteria were |log_2_ (fold change)| >1. As shown in **Figure 2C**, MMP1 was the only highly expressed gene in PDTC/ATC relative to PTC when we chose |log_2_ (fold change)| >2 as the cutoff criteria. Another 16 down-regulated genes were listed in **Figure 2D**, among which TG and TSHR are characteristic genes for PTC. In addition, MMP1 and other top 40 DEGs were exhibited in a heat map (**Figure 2E**).

Even if the above bulk RNA data from GEO showed MMP1 as a significantly upregulated gene in PDTC/ATC, MMP1 can be secreted by many cells including immune cells and stromal cells. To illustrate the expression of MMP1 in different cells, we used single-cell sequencing data in five ATC and six PTC patients (**Figure 2F**). After analyzing a total of 32168 cells, we found 29 clusters of different cells (**Supplementary Figure 1B**) and defined all cells as four basic types (**Figure 2G**). MMP1 was expressed mostly in tumor cells but rarely in other cells as shown in **Figure 2H**. Comparing its expression in PTC and ATC tumor cells, we found that MMP1 showed major expression in ATC tumor cells but lower expression in PTC tumor cells (**Figure 2I**). This reminds us that MMP1 expression in tumor cells might be related to the heterogeneity of different TC cells, and MMP1 was upregulated in ATC cells.

### Clinical significance of MMP1 in PTC and PDTC/ATC

**Figure 3:**
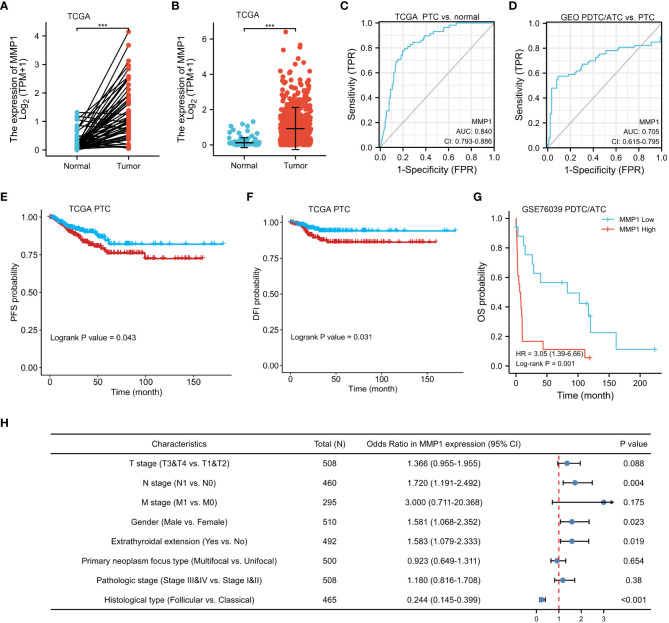
Clinical significance of MMP1 in PTC and PDTC/ATC. **(A)** The mRNA levels of MMP1 were up-regulated in PTC samples, which were downloaded from the TCGA database containing 58 paired PTC and normal tissue samples. **(B)** MMP1 was up-regulated in PTC samples, which were downloaded from the TCGA database containing 510 PTC samples and 58 normal tissue samples. **(C)** MMP1 effectively discriminated between PTC and normal tissues from the TCGA database. **(D)** MMP1 effectively discriminated between PDTC/ATC and PTC tissues from the GEO database. PTC patients with higher MMP1 levels harbor worse **(E)** PFS and **(F)** DFI. PDTC/ATC patients with higher MMP1 levels harbor worse **(G)** OS. **(H)** Forest plot of MMP1 in univariate logistic regression analyses of clinicopathological characteristics of the PTC patients from TCGA database. GEO, the Gene Expression Omnibus; PDTC, poorly differentiated thyroid cancer; ATC, anaplastic thyroid cancer; PTC, papillary thyroid cancer; TCGA, The Cancer Genome Atlas; AUC, the area under the curve; CI, confidence interval; vs., versus; HR, hazard ratio; PFS, progression-free survival; DFI, disease-free interval; OS, overall survival. ***P <0.001.

Data of MMP1 mRNA expression levels in PTC tissues and normal thyroid tissues were obtained from TCGA to demonstrate the role of MMP1 expression in PTC tumorigenesis. As shown in **Figures 3A, B**, MMP1 expression in PTC tumor tissues was significantly higher than that in normal thyroid tissues, whether they were paired samples or not. ROC curves were plotted to investigate the diagnostic value of MMP1, and the area under the curve (AUC) was 0.840 (**Figure 3C**) and 0.705 (**Figure 3D**) respectively, which can be used to distinguish the normal tissues from PTC, as well as PTC from PDTC/ATC, respectively. The clinicopathological characteristics of PTC patients from TCGA were summarized in **Table 1**. MMP1 expression was significantly associated with gender (P=0.029), T stage (P =0.004), N stage (P =0.005), extrathyroidal extension (P =0.025), histological type (P <0.001) and the thyroid gland disorder history (P <0.001).

**Table 1:** Clinical characteristics of PTC patients from TCGA database according to MMP1 low or high expression.

Characteristics	Low expression of MMP1 (n=255)	High expression of MMP1 (n=255)	P-value
Age, median (IQR)	48 (36, 58)	46 (34, 58)	0.597
Gender, n (%)			**0.029**
Female	197 (38.6%)	174 (34.1%)	
Male	58 (11.4%)	81 (15.9%)	
T stage, n (%)			**0.004**
T1	83 (16.3%)	60 (11.8%)	
T2	82 (16.1%)	85 (16.7%)	
T3	86 (16.9%)	89 (17.5%)	
T4	4 (0.8%)	19 (3.7%)	
N stage, n (%)			**0.005**
N0	127 (27.6%)	102 (22.2%)	
N1	97 (21.1%)	134 (29.1%)	
M stage, n (%)			0.190
M0	132 (44.7%)	154 (52.2%)	
M1	2 (0.7%)	7 (2.4%)	
Pathologic stage, n (%)			0.291
Stage I	144 (28.3%)	142 (28%)	
Stage II	29 (5.7%)	23 (4.5%)	
Stage III	58 (11.4%)	55 (10.8%)	
Stage IV	22 (4.3%)	35 (6.9%)	
Extrathyroidal extension, n (%)			**0.025**
No	179 (36.4%)	159 (32.3%)	
Yes	64 (13%)	90 (18.3%)	
Primary neoplasm focus type, n (%)			0.720
Multifocal	119 (23.8%)	114 (22.8%)	
Unifocal	131 (26.2%)	136 (27.2%)	
Histological type, n (%)			**< 0.001**
Classical	160 (31.4%)	204 (40%)	
Follicular	77 (15.1%)	24 (4.7%)	
Other	4 (0.8%)	5 (1%)	
Tall Cell	14 (2.7%)	22 (4.3%)	
Thyroid gland disorder history, n (%)			**< 0.001**
Lymphocytic Thyroiditis	47 (10.4%)	27 (6%)	
Nodular Hyperplasia	47 (10.4%)	21 (4.6%)	
Normal	119 (26.3%)	166 (36.7%)	
Other, specify	12 (2.7%)	13 (2.9%)	

Bold values show P < 0.05.

PTC, papillary thyroid cancer; TCGA, The Cancer Genome Atlas; IQR, interquartile range.

Kaplan-Meier survival analysis with a log-rank test was applied to determine the association between patients’ survival and MMP1 expression. As shown in **Figures 3E, F**, the MMP1 low-expressing group had significantly longer PFS and DFI in PTC patients from TCGA (P =0.043 and 0.031, respectively). Additionally, we collected OS data of 17 PDTC patients and 20 ATC patients from the GSE76039 dataset, and the prognosis of the MMP1 low-expressing group was still significantly better (**Figure 3G**, P =0.001), while the level of MMP1 in ATC is significantly higher than that in PDTC (8.24 versus 3.90, P <0.01). Next, univariate logistic regression analyses were performed to explore the relationship between MMP1 and some clinicopathological characteristics of the PTC patients from TCGA (**Figure 3H**), and MMP1 was positively related with N stage (OR =1.720, 95% CI = 1.191-2.492, P =0.004), and extrathyroidal extension (OR =1.583, 95% CI =1.079-2.033, P =0.023), respectively. The HRs for DFI of PTC patients from TCGA were explored to investigate the survival significance of MMP1. Only features with P <0.1 in univariate Cox regression were included in multivariate regression analysis. M1 stage, Pathologic stage III/IV, and high MMP1 were all confirmed to be independent risk factors for DFI in PTC patients (**Table 2**).

**Table 2:** Risk factors for DFI of PTC patients from the TCGA database.

Characteristics	Total (n)	Univariate analysis		Multivariate analysis	
		Hazard ratio (95% CI)	P-value	Hazard ratio (95% CI)	P-value
Gender	510				
Female	371	Reference			
Male	139	1.694 (0.975-2.945)	0.062	1.228 (0.553-2.730)	0.614
T stage	508				
T1&T2	310	Reference			
T3&T4	198	2.450 (1.417-4.236)	**0.001**	1.113 (0.306-4.042)	0.871
N stage	460				
N0	229	Reference			
N1	231	1.658 (0.936-2.934)	0.083	0.848 (0.380-1.892)	0.687
M stage	295				
M0	286	Reference			
M1	9	7.305 (2.780-19.197)	**<0.001**	4.366 (1.057-18.034)	**0.022**
Pathologic stage	508				
Stage I&II	338	Reference			
Stage III&IV	170	2.597 (1.520-4.437)	**<0.001**	2.850 (1.097-7.405)	**0.032**
Histological type	465				
Classical	364	Reference			
Follicular	101	0.574 (0.243-1.355)	0.206		
Primary neoplasm focus type	500				
Unifocal	267	Reference			
Multifocal	233	1.025 (0.595-1.764)	0.929		
Extrathyroidal extension	492				
No	338	Reference			
Yes	154	1.874 (1.092-3.216)	**0.023**	1.019 (0.323-3.216)	0.974
MMP1	510				
Low	255	Reference			
High	255	1.650 (1.249-2.870)	**0.036**	1.196 (1.046-2.224)	**0.042**

Patients with unknown key data were excluded from this analysis, and the number of patients included in this analysis was shown in the table. Bold values show P < 0.05.

DFI, disease-free interval; PTC, papillary thyroid cancer; TCGA, The Cancer Genome Atlas; CI, confidence interval; IQR, interquartile range.

### Function enrichment analysis

**Figure 4:**
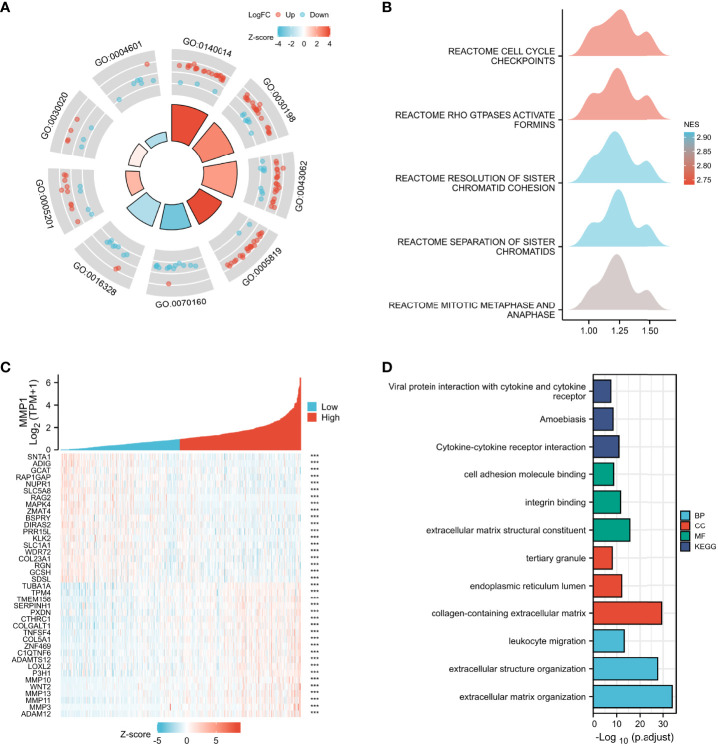
Enrichment analysis. GO **(A)** and GSEA **(B)** enrichment analyses of DEGs between PDTC/ATC and PTC samples. **(C)** Co-expression heatmap of top 10 positively associated genes and top 10 negatively associated genes with MMP1 form TGGA PTC samples. **(D)** GO and KEGG enrichment analyses of main associated genes of MMP1. GEO, the Gene Expression Omnibus; PDTC, poorly differentiated thyroid cancer; ATC, anaplastic thyroid cancer; PTC, papillary thyroid cancer; TCGA, The Cancer Genome Atlas; GO, Gene Ontology; GSEA, Gene set enrichment analysis; KEGG, Kyoto Encyclopedia of Genes and Genomes; NES, normalized enrichment score; BP, Biological Process; CC, Cellular Component; MF, Molecular Function; ***P <0.001.

Multiple functional enrichment analyses were performed using the GEO cohort and TCGA cohort to explore the different pathogenesis involved in this study. GO enrichment analysis (**Figure 4A**) indicated that the DEGs between PDTC/ATC and PTC samples from the GEO cohort were mostly enriched in extracellular matrix-related pathways (Details in **Supplementary Table 2**), which were reported to be significantly associated with the MMPs family (44), including MMP1. GSEA of the DEGs was also performed (**Figure 4B**), and the top 5 pathways between PDTC/ATC and PTC were cell cycle checkpoints (normalized enrichment score (NES) =2.73, P <0.01), GTPases active formins (NES= 2.73, P <0.01), resolution of sister chromatid cohesion (NES= 2.92, P <0.01), separation of sister chromatids (NES= 2.92, P <0.01), and mitotic metaphase and anaphase (NES= 2.84, P <0.01) (45) (Details in **Supplementary Table 3**). To more precisely explore the pathway associated with MMP1, we analyzed genes associated with MMP1 expression from the TCGA PTC samples, and the top 10 positively associated genes and top 10 negatively associated genes with MMP1 were shown in the co-expression heatmap (**Figure 4C**). Genes with |spearman correlation coefficient| >2 and p <0.05 were included in the subsequent GO and KEGG analysis, and the representative pathways are shown in **Figure 4D** (Details in **Supplementary Table 4**).

### Immune infiltration analysis

**Figure 5:**
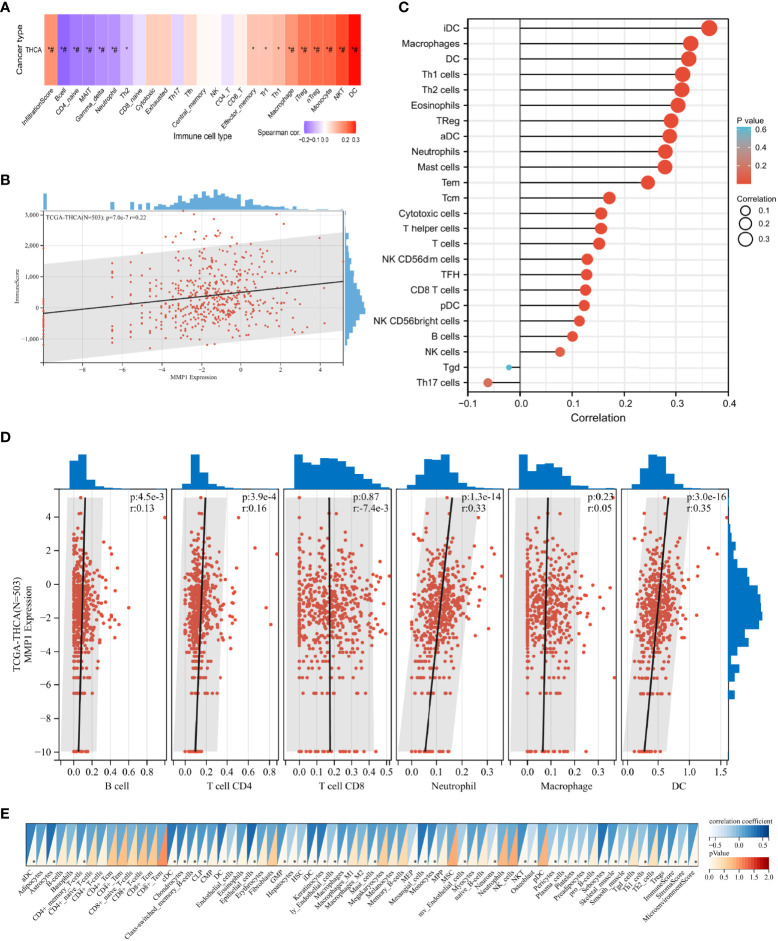
Correlation analysis of the expression of MMP1 with complex immune infiltration level in PTC samples from TCGA. **(A)** The correlation-heatmap between the GSVA scores and immune cell infiltrates over the PTC samples; **(B)** Correlation scatters plot of MMP1 levels and ImmuneScore of PTC samples. Different analyses of the correlation between MMP1 levels and immune infiltration: ssGSEA **(C)**, TIMER **(D)**, and xCELL **(E)**. PTC, papillary thyroid cancer; THCA, thyroid cancer. TCGA, The Cancer Genome Atlas; *P <0.05. ^#^ false discovery rate <0.05.

Immune infiltration in the tumor is a complex microenvironment that interacts with tumorigenesis, progression, and metastasis (46). We further explored the infiltration of immune cells in PTC, and the correlation between the GSVA scores and immune cell infiltrates over the PTC samples from TCGA is shown in **Figure 5A**. In addition, we explored the relationship between infiltrating immune cells and MMP1 expression levels. ImmuneScore is an indicator to characterize the immune landscape, and it was reported to be significantly correlated with PTC progression and dedifferentiation (47). MMP1 was identified to be positively correlated with ImmuneScore in this study (**Figure 5B**), which suggested that MMP1 might be involved in immune infiltration. ssGSEA (**Figure 5C**), TIMER (**Figure 5D**), and xCELL (**Figure 5E**) were then used to explore specific immune cells associated with MMP1, and dendritic cells (DCs), macrophages, and neutrophils were immune cells confirmed to be significantly associated with MMP1 in PTC *via* the 3 algorithms in this study. As shown in **Figures 5A, C, D**, Treg cells were also found to be significantly positively correlated with MMP1. Taken together, the above results demonstrated that MMP1 might influence the infiltration of immune cells in the PTC microenvironment.

### Analysis of the mutation, stemness, and differentiation

**Figure 6:**
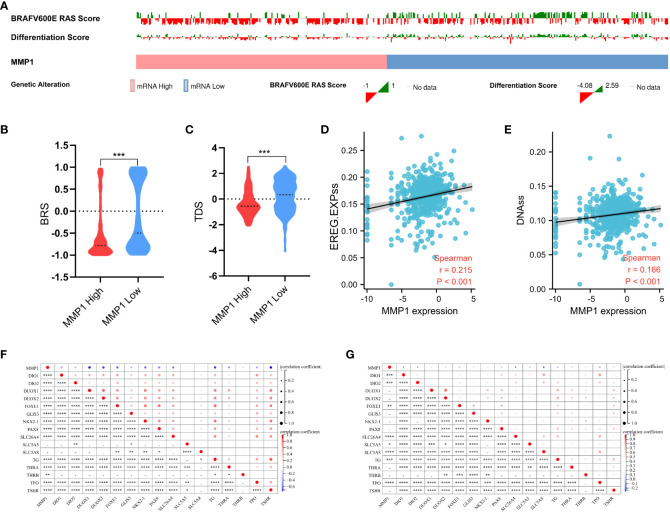
Analysis of the mutation, stemness, and differentiation. **(A)** Landscape of *BRAF* ^V600E^-*RAS* score and thyroid differentiation score based on MMP1 levels in PTC samples from TCGA. MMP1 high group had higher BRS **(B)** and lower TDS **(C)**. MMP1 was significantly positively correlated with the PTC stemness *via* both EREG.EXPss method **(D)** and DNAss method **(E)**. MMP1 was significantly negatively correlated with most TDS genes in both the GEO cohort of PDTC/ATC and PTC **(F)** and TCGA cohort of PTC **(G)**. GEO, the Gene Expression Omnibus; PDTC, poorly differentiated thyroid cancer; ATC, anaplastic thyroid cancer; PTC, papillary thyroid cancer; TCGA, The Cancer Genome Atlas; BRS, *BRAF* ^V600E^-*RAS* score; TDS, thyroid differentiation score. *P <0.05, **P <0.01, ***p <0.001, and ****p <0.0001.

The mutation indicators BRS of PTC samples from TCGA was displayed in **Figures 6A, B**, and the higher MMP1, the lower BRS, which means the high MMP1 group had a higher propensity for *BRAF*-like mutations. In other words, MMP1 may be involved in the *BRAF* ^V600E^- or *RAS*-mutant profiles in PTC. The stemness (self-renewal, differentiation, and fate determination) of a tumor could participate in multi-step tumorigenesis, recurrence, and metastasis (48). We quantified the stemness level of PTC samples from TCGA and explored its relationship with MMP1. The expression level of MMP1 was significantly positively correlated with the PTC stemness *via* both EREG.EXPss method (**Figure 6D**) and DNAss method (**Figure 6E**). As for the differentiation indicator, we used TDS to assess the differentiation level of PTC samples from TCGA. The results revealed that the higher MMP1 expression group had the lower TDS (**Figure 6C**). Specific associations between MMP1 and the 16 TDS-related genes were also explored, and the expression levels of MMP1 and most TDS genes were negatively correlated in both the GEO cohort of PDTC/ATC and PTC (**Figure 6F**) and TCGA cohort of PTC (**Figure 6G**). Given the above, MMP1 was considered to be involved in the dedifferentiation of PTC and could serve as a potential indicator for the differentiation level.

### Exploration of MMP1 in cell lines

**Figure 7:**
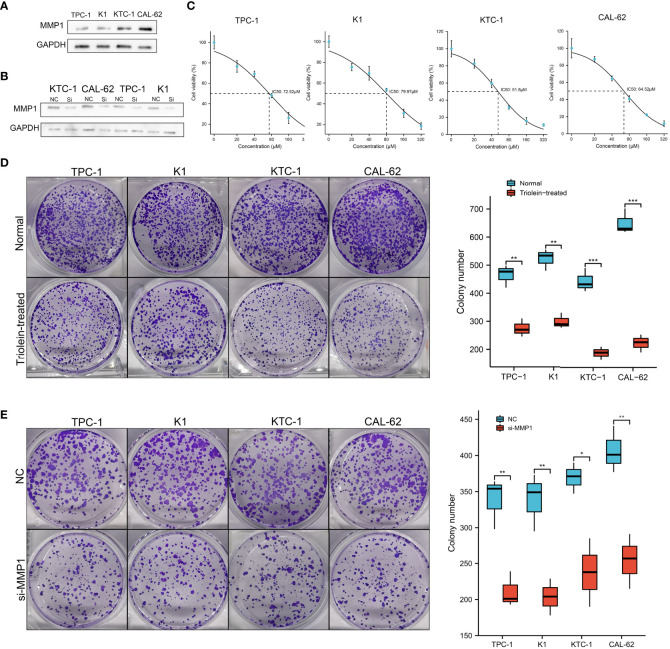
Exploration of MMP1 *via in vitro* experiments. **(A)** MMP1 was up-regulated in PDTC cell line KTC-1 and ATC cell line CAL-62. **(B)** MMP1 was significantly down-regulated in cells after transfection with MMP1 siRNA detected by western blot. **(C)** IC_50_ of triolein in different cell lines. Triolein **(D)** and MMP1 knockdown **(E)** inhibited colony formation in different cell lines. PDTC, poorly differentiated thyroid cancer; ATC, anaplastic thyroid cancer; PTC, papillary thyroid cancer; IC_50_, half-maximal inhibitory concentration. *P <0.05, **P <0.01, and ***p <0.001.

Since our findings above were based on RNA sequencing data obtained from public databases, we further explored and verified the expression and function of MMP1 in cell lines. PTC cell lines TPC-1 and K1, PDTC cell line KTC-1, and ATC cell line CAL-62 were used to perform western blotting assays. As shown in **Figure 7A**, high levels of MMP1 protein were detected in KTC-1 and CAL-62, especially in the ATC cell line CAL-62. Exogenous introduction of MMP1 siRNA was used to knock down the expressing levels of MMP1 in TPC-1, K1, KTC-1, and CAL-62 (**Figure 7B**). To further explore the potential of MMP1 as a therapeutic target in PDTC/ATC or PTC, a specific inhibitor triolein for MMP1 was used in this study. CCK8 assays were performed to determine cell viability in cell lines treated with different concentrations of triolein, and the half-maximal inhibitory concentrations (IC_50_) of triolein in different cell lines were also detected. The IC_50_ of triolein was 72.52 μM for TPC-1, 79.97 μM for K1, 51.80 μM for KTC-1, and 64.52 μM for CAL-62 (**Figure 7C**), respectively. Colony formation assays were also performed to analyze the cell viability of tumor cells under triolein treatment and MMP1 knockdown, where we observed that triolein and MMP1 knockdown both inhibited cell proliferation of all the TC cell lines (**Figures 7D, E**). Collectively, these results provide us with robust evidence indicating that MMP1 may serve as a novel biomarker and therapeutic target for PDTC/ATC and probably as a potential therapeutic target for PTC with the risk of progression or dedifferentiation.

## Discussion

The progression and dedifferentiation of PTC greatly affect the prognosis of patients (7), thus early detection and timely intervention for aggressive PTC are very necessary. In this study, we used transcriptome data of two cohorts, the GEO cohort of PDTC/ATC and PTC, and the TCGA cohort of PTC to perform differential analysis and functional annotation to evaluate the role of MMP1 as a potential regulator of tumor progression and dedifferentiation in PTC. Comprehensive and detailed assessments for the association between MMP1 and PTC clinicopathologic characteristics, survival, function, immune microenvironment, mutation, stemness, and dedifferentiation were conducted. In addition, our *in vitro* functional experiments preliminarily explored the effect of MMP1 inhibitor triolein on PTC and provided a potential therapeutic target for the treatment of aggressive TC, especially PDTC and ATC.

MMP1 along with other members of MMPs is a proteolytic enzyme that degrades multiple components of the extracellular matrix (49). The catalysis activity of MMP1 on fibrils depends on the random motion and substrate properties as well as on active site catalysis and conformational dynamics applicable to both fibrils and monomers (50). In this study, we only focused on the total forms of MMP1. In addition to playing a role in extracellular matrix transformation, MMPs participate in tumor progression by regulating signaling pathways that control cell growth, inflammation, or angiogenesis, and may even act in a non-proteolytic role (51, 52). A previous study (53) tried to determine the usefulness of MMP1 for differential diagnosis of follicular thyroid lesions, particularly between minimally invasive carcinoma and adenoma. However, no discriminative effect of MMP1 was found. The tumor-promoting roles of MMP1 have been reported in multiple cancers, including ovarian cancer (54), pancreatic cancer (55), and liver cancer (56), et al. In previous reports, MMP1 was identified to be associated with higher clinical stages of PTC (16–18), and MMP1 was overexpressed in ATC upon microarray analysis (19).

In our study, further analysis indicated that MMP1 might play an important role in the progression and dedifferentiation in PTC and PDTC/ATC, and as far as we knew, it was the first study to explore the role of MMP1 in these two groups. Both bulk and single-cell sequencing data indicated that MMP1 was high-expressed in PDTC/ATC. The ROC analysis proved the excellent diagnostic efficacy of MMP1 in predicting PTC and PDTC/ATC, and survival analysis also demonstrated its role in predicting the PFS/DFI in PTC patients and OS in ATC/PDTC patients. Due to the limited sample size and data sources, we pooled ATC and PDTC from GSE76039 together to analyze the correlation between their prognosis and MMP1 level instead of analyzing them separately. However, it is worth noting that since MMP1 levels are significantly higher in ATC than in PDTC, there is a possibility that the difference in survival is more related to tumor type than MMP1 expression, which should be explored by further studies. Both Logistic and Cox regressions confirmed the correlations between MMP1 and worse outcomes for PTC patients. Enrichment analysis of our study demonstrated that extracellular matrix-related pathways were significantly elevated in PDTC/ATC compared with PTC, while MMP1 played important roles in these pathways.

The associations between immune infiltration and PTC progression and dedifferentiation have long been demonstrated (47, 57). DCs, macrophages, neutrophils, and Treg cells are important components in the immune microenvironment of PTC and have been proven to promote tumor progression and poor prognosis (58–60). In our study, MMP1 was found to be positively associated with the above immune cells in PTC, which demonstrated that MMP1 might promote PTC progression by interacting with the tumor-associated immune infiltration. Our study also found other immune cells, such as NK cells and monocytes, significantly associated with MMP1 in PTC *via* different immune cell function analyses in different ways, detailed mechanisms should be confirmed by further studies.

Gene mutations, including *BRAF* ^V600E^, *RAS*, and *TERT* promoter mutations, exert extensive effects on oncogenic signaling pathways in PTC and PDTC/ATC (61). The expression level of MMP1 was also significantly correlated with the mutation indicator BRS in our study. As for stemness and differentiation, they are usually analyzed together. There is homeostasis between stemness and differentiation, and the stronger the stemness, the higher potential of the differentiation, or in the other words, the more poorly differentiated (62). In this study, a positive correlation was found between MMP1 and stemness, and a negative correlation was found between MMP1 and the differentiation level of PTC, which strongly suggested that MMP1 might promote the dedifferentiation. Since MMP1 was also correlated with many differentiation and dedifferentiation markers of thyroid, such as TDS-related genes, MAPK4, SERPINE1, LOXL2, COL5A1, et al., the potential interactions between them require further investigations to verify the above findings. MMPs are thought to be ideal therapeutic targets for cancer as more and more novel, highly selective, and potent MMP inhibitors are now available (63). SD-7300, an oral inhibitor of MMP-2, -9, and -13, has shown promising preclinical therapeutic effects for breast cancer (64). Triolein and its analogs have been shown to have some antitumor effects in other tumors (65, 66). In this study, we preliminarily verified the inhibitory effect of the MMP1 inhibitor triolein on TC cells, especially on PDTC and ATC.

To the best of our knowledge, the current study is the first to comprehensively disclose that MMP1 may be a potential regulator of tumor progression and dedifferentiation in PTC. Furthermore, we also explored the role of MMP1 in PTC clinicopathologic characteristics, survival, function, immune microenvironment, mutation, stemness, and dedifferentiation. The *in vitro* functional experiments in this study further proved that MMP1 could be used as a potential therapeutic target for aggressive TC. However, there are still some limitations to the present study. First of all, a large number of findings and results were based on data from public databases, and some detailed data were not available, which might cause some bias. Second, we have not thoroughly explored the underlying mechanisms by which MMP1 exerts its tumor-promoting effects in PTC. Finally, we only validated the function of MMP1 and its inhibitor triolein *via in vitro* experiments, while *in vivo* experiments may give us more insight into this topic.

## Conclusion

In conclusion, the integrated bioinformatic analysis revealed that MMP1 might act as a regulator for tumor progression and dedifferentiation in PTC, which was confirmed *via* the *in vitro* experiments. Our findings suggested that MMP1 could be a potential biomarker and therapeutic target for PTC, especially for the aggressive PTC that might dedifferentiate into PDTC or ATC.

## Data availability statement

The original contributions presented in the study are included in the article/**Supplementary Material**. Further inquiries can be directed to the corresponding authors.

## Author contributions

TH conceived, designed, and supervised the study with FD, JZ, and MX. JZ and MX collected and analyzed data, and wrote the draft of the manuscript. JT, LZ, and FD analyzed the data and reviewed the manuscript. TH and FD oversaw the bioinformatics data analyses and modified and improved the manuscript. All authors contributed to the article and approved the submitted version.

## Funding

This study is supported by the National Natural Science Foundation of China (Grant 82002834) and Key Program of Natural Science Foundation of Hubei Province (Grant No. 2021BCA142).

## Acknowledgments

Thanks to all the members who assisted in this study. Thanks for the guidance of the Sangerbox group on bioinformatics.

## Conflict of interest

The authors declare that the research was conducted in the absence of any commercial or financial relationships that could be construed as a potential conflict of interest.

## Publisher’s note

All claims expressed in this article are solely those of the authors and do not necessarily represent those of their affiliated organizations, or those of the publisher, the editors and the reviewers. Any product that may be evaluated in this article, or claim that may be made by its manufacturer, is not guaranteed or endorsed by the publisher.

## Supplementary material

Supplementary Figure 1
**(A)** Cell markers. **(B)** 29 clusters of different cells form ATC and PTC.
