# Supplementary figures and images for: MMP1 acts as a potential regulator of tumor progression and dedifferentiation in papillary thyroid cancer
Source: Front Oncol. 2022 Nov 21;12:1030590. doi: 10.3389/fonc.2022.1030590 (PMC9720150; doi:10.3389/fonc.2022.1030590)

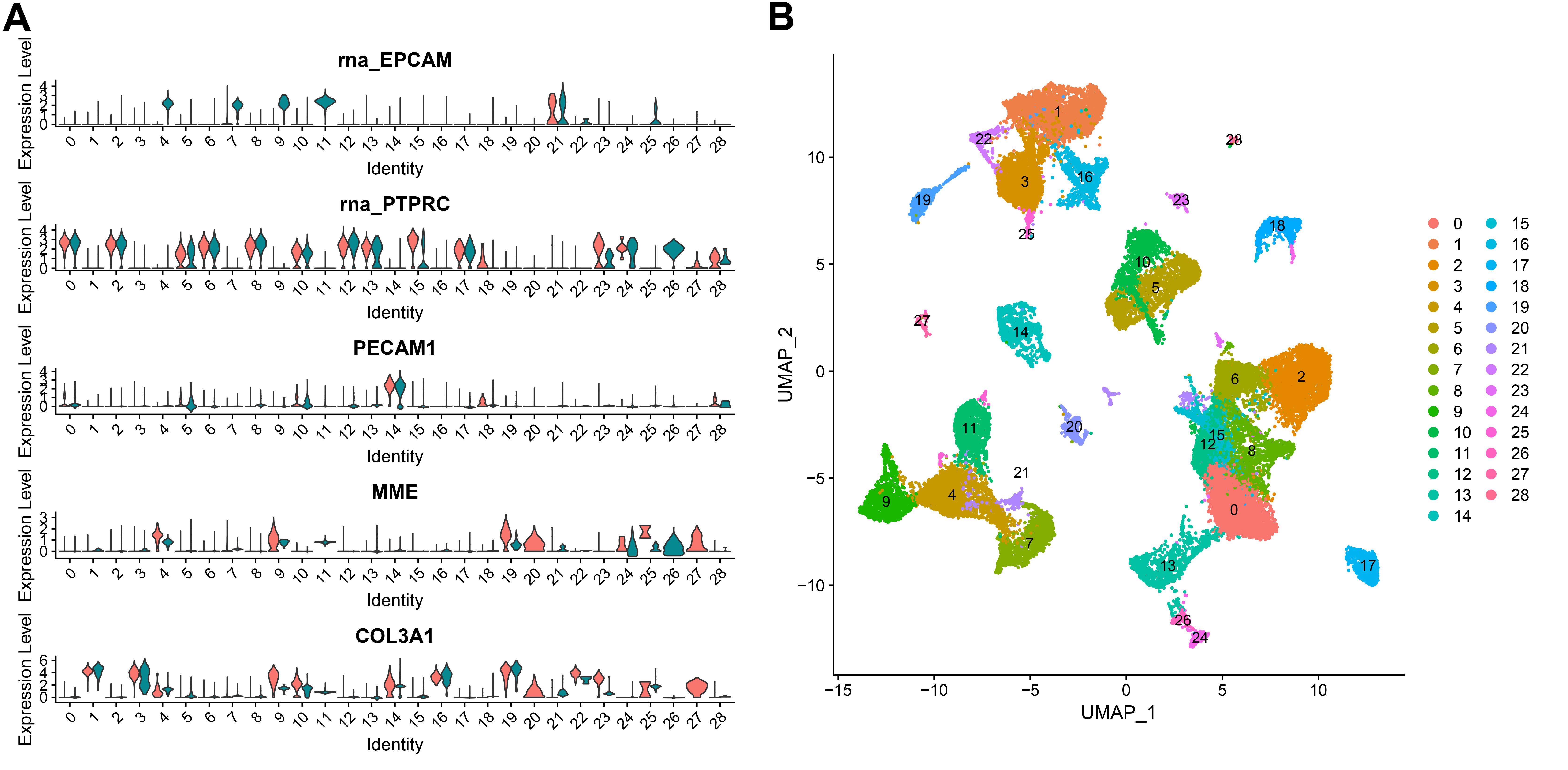

Supplement: Supplementary Figure 1 — (A) Cell markers. (B) 29 clusters of different cells form ATC and PTC. [file Image_1.tif]
